# Remdesivir-Associated Survival Outcomes Among Immunocompromised Patients Hospitalized for COVID-19: Real-world Evidence From the Omicron-Dominant Era

**DOI:** 10.1093/cid/ciae510

**Published:** 2024-10-15

**Authors:** Essy Mozaffari, Aastha Chandak, Robert L Gottlieb, Chidinma Chima-Melton, Mark Berry, Alpesh N Amin, Paul E Sax, Andre C Kalil

**Affiliations:** Medical Affairs, Gilead Sciences, Foster City, California, USA; Evidence and Access, Certara, New York, New York, USA; Department of Internal Medicine, Baylor University Medical Center, Dallas, Texas, USA; Baylor Scott & White Heart and Vascular Hospital, Dallas, Texas, USA; Baylor Scott & White The Heart Hospital, Plano, Texas, USA; Baylor Scott & White Research Institute, Dallas, Texas, USA; Pulmonary Division, Tele-ICU, Los Angeles, California, USA; Medical Affairs, Gilead Sciences, Foster City, California, USA; Department of Medicine, School of Medicine, University of California Irvine, Irvine, California, USA; Division of Infectious Diseases, Brigham and Women's Hospital, Boston, Massachusetts, USA; Division of Infectious Diseases, Department of Internal Medicine, University of Nebraska Medical Center, Omaha, Nebraska, USA

**Keywords:** remdesivir, immunocompromised, COVID-19, SARS-CoV-2, transplantation, cancer, hematological malignancy, leukemia, lymphoma, multiple myeloma, real-world data, data science, propensity score, comorbidity, omicron

## Abstract

**Background:**

Patients with immunocompromising conditions are at increased risk for coronavirus disease 2019 (COVID-19)–related hospitalizations and deaths. Randomized clinical trials provide limited enrollment, if any, to provide information on the outcomes in such patients treated with remdesivir.

**Methods:**

Using the US PINC AI Healthcare Database, we identified adult patients with immunocompromising conditions, hospitalized for COVID-19 between December 2021 and February 2024. The primary outcome was all-cause inpatient mortality examined in propensity score–matched patients in remdesivir vs nonremdesivir groups. Subgroup analyses were performed for patients with cancer, hematological malignancies, and solid organ or hematopoietic stem cell transplant recipients.

**Results:**

Of 28 966 patients included in the study, 16 730 (58%) received remdesivir during the first 2 days of hospitalization. After propensity score matching, 8822 patients in the remdesivir and 8822 patients in the nonremdesivir group were analyzed. Remdesivir was associated with a significantly lower mortality rate among patients with no supplemental oxygen (adjusted hazard ratio [95% confidence interval], 0.73 [.62–.86] at 14 days and 0.79 [.68–.91] at 28 days) and among those with supplemental oxygen (0.75 [.67–.85] and 0.78 [.70–.86], respectively). Remdesivir was also associated with lower mortality rates in subgroups of patients with cancer, hematological malignancies (leukemia, lymphoma, or multiple myeloma), and solid organ or hematopoietic stem cell transplants.

**Conclusions:**

In this large cohort of patients with immunocompromising conditions hospitalized for COVID-19, remdesivir was associated with significant improvement in survival, including patients with varied underlying immunocompromising conditions. The integration of current real-world evidence into clinical guideline recommendations can inform clinical communities to optimize treatment decisions in the evolving COVID-19 era, extending beyond the conclusion of the public health emergency declaration.

## Teaser

In the Omicron era, remdesivir continues to be associated with significant improvement in survival among patients with immunocompromising conditions hospitalized for coronavirus disease 2019. The benefits extend to different types of immunocompromising conditions.

Immunocompromised patients continue to experience >2-fold risk for hospitalizations for coronavirus disease 2019 (COVID-19) and increased rates of breakthrough infections and death compared with immunocompetent patients [1–6]. Patients with immunocompromising conditions represent about 6.2% of the US population [7]. These conditions include cancer, human immunodeficiency virus (HIV) infection, transplantation, primary immunodeficiency, and treatment with immunosuppressive medications [8, 9].

When hospitalized, immunocompromised patients remain at a high risk for COVID-19–related severe outcomes, intensive care unit admission, and death [10–12]. A retrospective study of close to 12 million individuals revealed that immunocompromised patients comprised about 4% of the study population but disproportionally accounted for 22% of hospitalizations, 28% of intensive care unit admissions, and 24% of deaths, all COVID-19 related [12].

Among immunocompromised patients, the risk of COVID-19–related hospitalization or death is highest among transplant recipients, with a 23-fold higher risk in solid organ transplant recipients (SOTRs) and a >6-fold higher risk in hematopoietic stem cell transplant (HSCT) recipients compared with patients who are not transplant recipients [1, 13]. Compared with those without cancer, patients with cancer have an almost 3-fold higher risk of COVID-19–related death [1]. Given these risk profiles for different immunocompromising conditions, additional data are needed to address appropriate treatments of COVID-19 in patients with these conditions to improve survival.

The treatment of COVID-19 in immunocompromised patients presents several challenges. Besides impaired response to COVID-19 vaccinations, immunocompromised patients may experience impaired immune response, prolonged shedding of severe acute respiratory syndrome coronavirus 2 (SARS-CoV-2), and an increased risk for developing symptomatic COVID-19 [8, 14, 15]. Patients with immunocompromising conditions with prolonged COVID-19 infection may develop individual mutations and may contribute to the creation of additional SARS-CoV-2 variants spreading in the general community [16, 17].

Although numerous guidelines are available for managing COVID-19 during hospitalizations, few address the treatment of immunocompromised patients. The recently retired guideline from the National Institutes of Health (NIH) recommended using remdesivir in patients who are immunocompromised and hospitalized for COVID-19 [9]. Other guidelines provide limited guidance, if any, for the management of immunocompromised patients hospitalized for COVID-19 [18–21].

Remdesivir, a SARS-CoV-2 nucleotide analogue RNA polymerase inhibitor, is indicated for treatment of COVID-19 in hospitalized and nonhospitalized patients [22]. The use of remdesivir in hospitalized immunocompromised patients is primarily supported by observational studies [8, 23]. During early stages of the COVID-19 pandemic, few randomized clinical trials included immunocompromised patients, and even in those that did, the sample sizes of immunocompromised patients were frequently too small to allow for appropriate subgroup analyses [24]. For example, immunocompromised patients represented close to 10% of enrolled patients in the phase 3 randomized DisCoVeRY trial of remdesivir, but their outcomes were not reported specifically [25]. The reasons for excluding immunocompromised patients from randomized trials, especially those who were severely immunocompromised, included concerns about the potential impact of confounding medical complexity, altered pharmacokinetics, and different outcomes [24].

More recently, emerging evidence from real-world data has provided additional insight regarding the appropriate management of immunocompromised patients hospitalized for COVID-19. A retrospective comparative effectiveness study in 30 397 patients with immunocompromising conditions hospitalized for COVID-19 revealed an approximate 20%–30% reduction in 14- and 28-day mortality rates associated with the early use of remdesivir [26]. The results of this study have been incorporated into the NIH guideline sections that discussed the management of COVID-19 in patients who are immunocompromised [9]. Real-world evidence played a critical role in informing the recommendations for management of COVID-19 in immunocompromised patients due to the lack of evidence for this patient population in the randomized clinical trials.

Given the specific challenges in treating and vulnerability of patients with immunocompromising conditions, we aim to provide recent evidence on the outcomes of remdesivir use in this patient population, during the Omicron period, extending through early 2024 and covering almost 1 year beyond the end of the US public health emergency declaration. Results from our study will add to the existing evidence by providing current information on the effectiveness of remdesivir among immunocompromised patients, specifically with the evolving Omicron subvariants, which were not examined in prior study periods. Furthermore, use of a large national database allows us to analyze patients with specific conditions of interest, which may not be possible in the randomized clinical trials due to strict inclusion/exclusion criteria. Thus, the several motivations described above will provide relevant evidence to aid clinicians to optimize the treatment management of this highly vulnerable immunocompromised patient population.

## METHODS

### Study Design and Data Source

This retrospective comparative effectiveness study used records of hospitalized patients from the US PINC AI Healthcare Database (formerly Premier Healthcare Database; www.pinc-ai.com). The database, compliant with the Health Insurance Portability and Accountability Act, captures approximately 25% of hospitalizations across 48 states in the United States and includes patient-level demographic data, disease state, diagnoses at admission and discharge, and hospital characteristics, as well as billing data for day-level clinical activities, including procedures, devices, and medications. More than 99% of patient records in the data set are complete for all data elements recorded, and more than 99.99% are complete for key data elements, such as demographics and diagnostic information.

### Study Population

The study population consisted of adult patients (≥18 years old) with immunocompromising conditions who were hospitalized between 1 December 2021 and 29 February 2024 and had a primary discharge diagnosis of COVID-19 (*International Classification of Diseases, Tenth Revision, Clinical Modification* [*ICD-10-CM*] code U07.1) that was flagged as “present on admission.” Patients with immunocompromising conditions were identified using *ICD-10-CM* codes for immunocompromising conditions including cancer, solid organ transplant and HSCT, hematological malignancies, moderate or severe primary immunodeficiencies, immunosuppressive medications, asplenia, bone marrow failure or aplastic anemia, HIV, and toxic effects of antineoplastics (Supplementary Table 1).

Exclusion criteria included pregnancy, incomplete data fields in the hospital records, transfer from hospice or another hospital, transfer to another hospital, admission for an elective procedure, use of extracorporeal membrane oxygenation (ECMO) on admission, discharge or death during the first 2 days of hospitalization, or initiation of remdesivir after the first 2 days of hospitalization. Supplemental oxygen requirements were also assessed within the first 2 days. Patients admitted to hospitals that did not report separate charges for supplemental oxygen were excluded from this study. This step was performed to account for hospitals that include the charges for supplemental oxygen supply in the room charges instead of billing for them separately.

Patients were considered as treated with remdesivir (referred to as “patients receiving remdesivir”) if they received ≥1 dose of remdesivir within the first 2 calendar days of hospitalization for COVID-19. Patients were considered as not receiving remdesivir (“patients not receiving remdesivir”) if they did not receive remdesivir throughout their hospitalization for COVID-19. Patients who started remdesivir late after the baseline period were excluded from the primary analysis, because late initiation may represent salvage therapy or treatment after progression, which was not explored in the current study.

### Definition of Study Variables

The baseline was defined as the first 2 days of hospitalization. Baseline variables included demographic and clinical characteristics, hospital characteristics, hospital ward on admission, and baseline supplemental oxygen requirements. Baseline supplemental oxygen requirement was described as “no supplemental oxygen charges” (NSOc) in hospitals that charge for oxygen and the presence of any oxygen charges, including low-flow oxygen, high-flow oxygen/noninvasive ventilation, or invasive mechanical ventilation/ECMO. The study period was split into the earlier Omicron period (December 2021 to December 2022) and the later Omicron period (January 2023 to February 2024), based on the predominant SARS-CoV-2 variants in the United States [27, 28]. The US public health emergency expired on 11 May 2023 [29]. Remdesivir therapy duration was descriptively summarized for the study population and all the subgroups.

### Outcomes

The primary outcome was all-cause inpatient mortality at 14 and 28 days after baseline, defined as a discharge status of “expired” or “hospice.” Patients were followed up from day 3 of the hospitalization (ie, after the baseline period during which remdesivir treatment and other baseline supplemental oxygen requirements were ascertained) through death or the end of follow-up. Patients who were discharged alive were censored at 14 or 28 days after discharge for the respective primary mortality assessments.

### Statistical Analyses

Patients who received remdesivir were matched to those who did not, using a 1:1 preferential propensity score (PS)–matching approach without replacement with a caliper distance of 0.2 times the standard deviation of the logit of PS. The matching process parallels the methods discussed in our previous comparative effectiveness studies [26, 30–32] and in the companion supplement publication by Mozaffari et al [33]. Matching occurred within the same age group (18–49, 50–64, or ≥65 years) and admission month group (in 2–3-month blocks) in the same hospital as the first step or, for the remaining unmatched patients, at another hospital of the same bed size (<200, 200–499, or ≥500 beds).

Subgroup analyses were performed in patients with cancer (including hematological malignacies) or specifically among patients with hematological malignancies including leukemia, lymphoma, or multiple myeloma and in SOTRs and HSCT recipients for the main outcome of all-cause inpatient mortality (Supplementary Table 1). Subgroups of these patients were identified from the matched cohort and were not mutually exclusive, as some patients could have more than one immunocompromising condition.

Mortality rates were summarized as crude (unadjusted) proportions of deaths/discharge to hospice within 14 and 28 days after baseline in the matched cohort. Further, the association of remdesivir with inpatient mortality at 14 and 28 days was evaluated using Cox proportional hazard models separately for the 2 time points; adjusted hazard ratios (aHRs) and 95% confidence intervals (CIs) were derived. The proportional hazard assumption was met for each analysis as assessed through Kaplan-Meier curves (where the curves did not cross over) and log of negative log plot (which showed reasonably parallel lines that did not cross over). The models were adjusted for hospital-level cluster effects using robust sandwich variance estimator and key covariates of age (as a continuous variable), admission month, hospital ward on admission, and postbaseline time-varying COVID-19 treatment (corticosteroids, baricitinib, or tocilizumab). These additional adjustment variables were prespecified to account for any remaining residual confounding among these variables as they were identified to be key covariates affecting the study outcomes. All analyses were stratified by baseline NSOc vs all supplemental oxygen requirements.

Several sensitivity analyses were performed as part of the study. Inverse probability of treatment weighting (IPTW) was carried out as a sensitivity analysis to PS matching, where PS scores <0.05 and >0.95 were trimmed. A sensitivity analysis for the treatment group definition was performed by comparing patients who initiated remdesivir within 2 days of admission with those who did not (ie, patients who were never treated with remdesivir or were treated after the first 2 days of hospitalization). To account for potential improper documentation of charges in the NSOc group, a sensitivity analysis excluded patients with an admission diagnosis of hypoxemia or respiratory distress requiring critical care on admission.

## RESULTS

### Study Population

**Figure 1.**
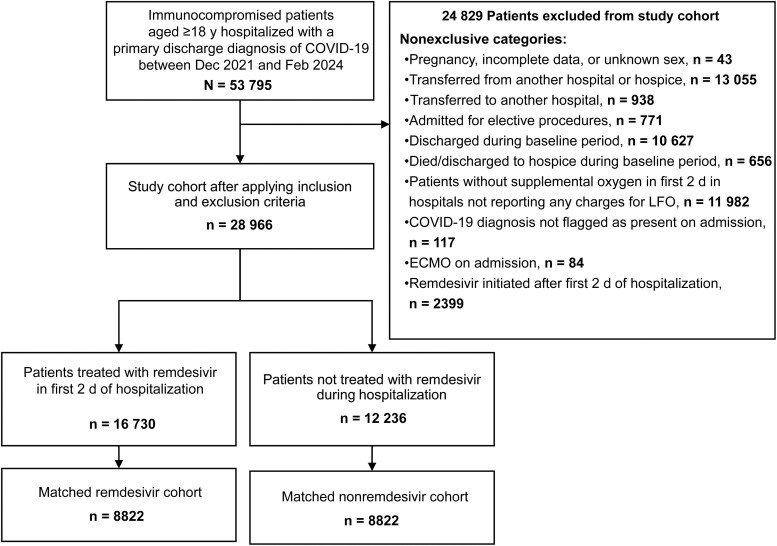
Study flow diagram. Abbreviations: COVID-19, coronavirus disease 2019; d, days; ECMO, extracorporeal membrane oxygenation; LFO, low-flow oxygen; y, years.

There were 53 795 adult patients with immunocompromising conditions hospitalized for COVID-19 within the specified study time frame (Figure 1). A total of 28 966 patients met the eligibility criteria, of whom 16 730 (58%) received remdesivir. After the 1:1 matching without replacement, the study cohort consisted of 8822 remdesivir and nonremdesivir matched pairs.

After matching, all baseline characteristics were well balanced with absolute standardized difference of <0.15 between the 2 groups (Table 1 and Supplementary Figure 1). Overall, in each cohort, most patients were ≥65 years old (77%), white (77%), and non-Hispanic (86%). Key comorbid conditions included cardiovascular disease (90%), cancer (43%), chronic obstructive pulmonary disease (39%), diabetes (38%), renal disease (37%), and obesity (25%). The most common qualifying immunocompromising conditions were cancer, including hematological malignancies (43% of matched patients in each group), receipt of immunosuppressive medications (35% in the nonremdesivir and 33% in the remdesivir group), and moderate or severe primary immunodeficiencies (26% and 31%, respectively). Furthermore, 84% of patients were admitted to the general hospital ward, and the rest to the intensive care or step-down unit. At baseline, most hospitalized patients received corticosteroids (79% of patients) and anticoagulants (71% of patients). At baseline, 54% of patients had NSOc, and supplemental oxygen requirements were low-flow oxygen in 30% of patients, high-flow oxygen/noninvasive ventilation in 15% of patients, and invasive mechanical ventilation/ECMO in 2% of patients. About two-thirds of the patients were hospitalized during the earlier Omicron period.

**Table 1.** Demographics of Patients With Immunocompromising Conditions Hospitalized for COVID-19 (December 2021 to February 2024)

Characteristic	Category	Patients, No. (%)			
		Before Matching		After Matching	
		Nonremdesivir (n = 12 236)	Remdesivir (n = 16 730)	Nonremdesivir (n = 8822)	Remdesivir (n = 8822)
Age group, y	18–49	825 (6.7)	1185 (7.1)	430 (4.9)	430 (4.9)
	50–64	2456 (20.1)	3489 (20.9)	1595 (18.1)	1595 (18.1)
	≥65	8955 (73.2)	12056 (72.1)	6797 (77.0)	6797 (77.0)
Gender	Female	6160 (50.3)	8587 (51.3)	4417 (50.1)	4507 (51.1)
Race	White	9258 (75.7)	12813 (76.6)	6791 (77.0)	6826 (77.4)
	Black	2002 (16.4)	2287 (13.7)	1310 (14.8)	1298 (14.7)
	Asian	192 (1.6)	390 (2.3)	154 (1.7)	144 (1.6)
	Other	784 (6.4)	1240 (7.4)	567 (6.4)	554 (6.3)
Ethnicity	Hispanic	928 (7.6)	1861 (11.1)	680 (7.7)	682 (7.7)
	Non-Hispanic	10408 (85.1)	13949 (83.4)	7565 (85.8)	7546 (85.5)
	Unknown	900 (7.4)	920 (5.5)	577 (6.5)	594 (6.7)
Primary payer	Commercial	1539 (12.6)	2418 (14.5)	1056 (12.0)	1055 (12.0)
	Medicare	9335 (76.3)	12478 (74.6)	6933 (78.6)	6917 (78.4)
	Medicaid	882 (7.2)	1297 (7.8)	549 (6.2)	566 (6.4)
	Other	480 (3.9)	537 (3.2)	284 (3.2)	284 (3.2)
Admission source	Transfer from skilled nursing or immediate care facility	366 (3.0)	543 (3.2)	274 (3.1)	290 (3.3)
Hospital size, no. of beds	<100	864 (7.1)	1128 (6.7)	623 (7.1)	625 (7.1)
	100–199	1936 (15.8)	2675 (16.0)	1306 (14.8)	1304 (14.8)
	200–299	2500 (20.4)	3109 (18.6)	1824 (20.7)	1816 (20.6)
	300–399	2302 (18.8)	2615 (15.6)	1631 (18.5)	1581 (17.9)
	400–499	1485 (12.1)	1875 (11.2)	1048 (11.9)	1106 (12.5)
	≥500	3149 (25.7)	5328 (31.8)	2390 (27.1)	2390 (27.1)
Hospital location	Urban	10796 (88.2)	15057 (90.0)	7876 (89.3)	7885 (89.4)
	Rural	1440 (11.8)	1673 (10.0)	946 (10.7)	937 (10.6)
Teaching hospital		5153 (42.1)	7747 (46.3)	3794 (43.0)	3751 (42.5)
Region	Midwest	3020 (24.7)	3881 (23.2)	2259 (25.6)	2179 (24.7)
	Northeast	1335 (10.9)	2644 (15.8)	1058 (12.0)	1067 (12.1)
	South	6618 (54.1)	8353 (49.9)	4582 (51.9)	4635 (52.5)
	West	1263 (10.3)	1852 (11.1)	923 (10.5)	941 (10.7)
Comorbid conditions	Obesity	3045 (24.9)	4342 (26.0)	2195 (24.9)	2242 (25.4)
	COPD	4561 (37.3)	6999 (41.8)	3472 (39.4)	3477 (39.4)
	Cardiovascular disease	11003 (89.9)	14825 (88.6)	7929 (89.9)	7956 (90.2)
	Diabetes	4730 (38.7)	6256 (37.4)	3341 (37.9)	3307 (37.5)
	Renal disease	4838 (39.5)	5269 (31.5)	3258 (36.9)	3237 (36.7)
	Cancer	5125 (41.9)	7163 (42.8)	3786 (42.9)	3781 (42.9)
Type of immunocompromising condition	Cancer	5125 (41.9)	7163 (42.8)	3786 (42.9)	3781 (42.9)
	Hematological malignancies	1865 (15.2)	2713 (16.2)	1390 (15.8)	1456 (16.5)
	Leukemia	797 (6.5)	1191 (7.1)	594 (6.7)	654 (7.4)
	Lymphoma	656 (5.4)	941 (5.6)	492 (5.6)	476 (5.4)
	Multiple myeloma	391 (3.2)	553 (3.3)	286 (3.2)	321 (3.6)
	SOTRs and HSCT recipients	888 (7.3)	1282 (7.7)	621 (7.0)	696 (7.9)
	Moderate or severe primary immunodeficiencies	3097 (25.3)	5144 (30.7)	2260 (25.6)	2732 (31.0)
	Immunosuppressive medications	4030 (32.9)	5992 (35.8)	3050 (34.6)	2936 (33.3)
	Asplenia	262 (2.1)	319 (1.9)	184 (2.1)	163 (1.8)
	Bone marrow failure/aplastic anemia	2037 (16.6)	2179 (13.0)	1269 (14.4)	1309 (14.8)
	HIV	208 (1.7)	291 (1.7)	111 (1.3)	117 (1.3)
	Toxic effects of antineoplastics	611 (5.0)	888 (5.3)	425 (4.8)	489 (5.5)
Hospital ward upon admission	General ward	10234 (83.6)	13452 (80.4)	7395 (83.8)	7389 (83.8)
	ICU/step-down unit	2002 (16.4)	3278 (19.6)	1427 (16.2)	1433 (16.2)
Admission diagnosis	Sepsis	69 (0.6)	77 (0.5)	41 (0.5)	40 (0.5)
	Pneumonia	771 (6.3)	1058 (6.3)	533 (6.0)	521 (5.9)
Other treatments at baseline	Anticoagulants	8114 (66.3)	12351 (73.8)	6224 (70.6)	6216 (70.5)
	Convalescent plasma	9 (0.1)	34 (0.2)	6 (0.1)	6 (0.1)
	Corticosteroids	8381 (68.5)	14287 (85.4)	6969 (79.0)	6993 (79.3)
	Baricitinib	476 (3.9)	616 (3.7)	349 (4.0)	342 (3.9)
	Tocilizumab	278 (2.3)	568 (3.4)	215 (2.4)	226 (2.6)
	Oral antivirals	224 (1.8)	41 (0.2)	12 (0.1)	19 (0.2)
Baseline supplemental oxygen requirements	NSOc	6786 (55.5)	7790 (46.6)	4774 (54.1)	4774 (54.1)
	LFO	3360 (27.5)	5391 (32.2)	2622 (29.7)	2622 (29.7)
	HFO/NIV	1721 (14.1)	3183 (19.0)	1278 (14.5)	1278 (14.5)
	IMV/ECMO	369 (3.0)	366 (2.2)	148 (1.7)	148 (1.7)
Omicron period	Earlier (Dec 2021–Dec 2022)	8505 (69.5)	11093 (66.3)	6251 (70.9)	6251 (70.9)
	Later (Jan 2023–Feb 2024)	3731 (30.5)	5637 (33.7)	2571 (29.1)	2571 (29.1)

Abbreviations: COPD, chronic obstructive pulmonary disease; COVID-19, coronavirus disease 2019; ECMO, extracorporeal membrane oxygenation; HFO, high-flow oxygen; HIV, human immunodeficiency virus; HSCT, hematopoietic stem cell transplant; ICU, intensive care unit; IMV, invasive mechanical ventilation; LFO, low-flow oxygen; NIV, noninvasive ventilation; NSOc, no supplemental oxygen charges; SOTRs solid organ transplant recipients; y, years.

### Outcomes

**Figure 2.**
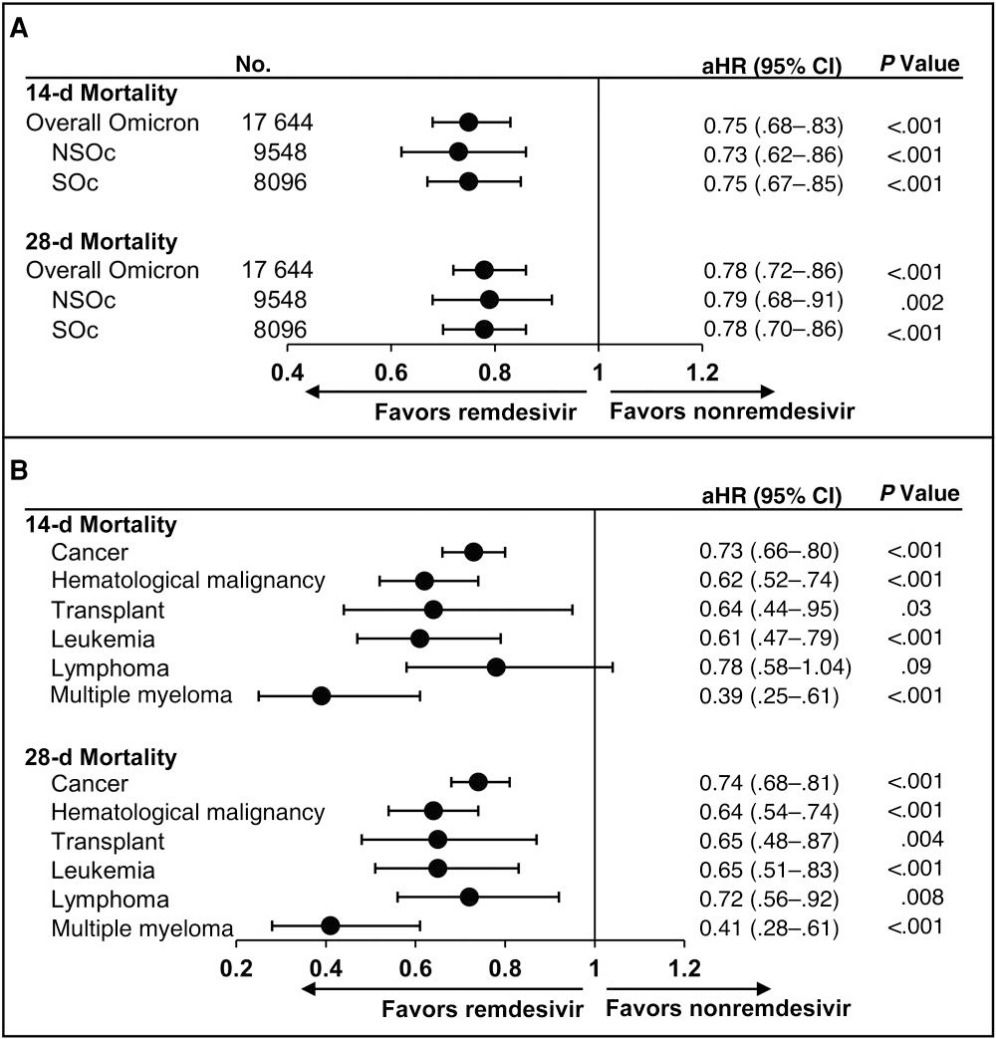
Mortality rates at 14 and 28 days in all patients with immunocompromising conditions (*A*) and by patient subgroup (cancer, hematological malignancies, transplant [solid organ or hematopoietic stem cell], leukemia, lymphoma, and multiple myeloma) (*B*). Cox proportional hazards models were used to derive estimates adjusted for age, admission month, hospital ward on admission (intensive care unit vs general ward), and time-varying treatment with other COVID-19 medications (baricitinib, tocilizumab, and oral antivirals). Abbreviations: aHR, adjusted hazard ratio; CI, confidence interval; COVID-19, coronavirus disease 2019; d, day; NSOc, no supplemental oxygen charges; SOc, supplemental oxygen charges.

The unadjusted all-cause inpatient mortality risk was 9.2% in patients who received remdesivir and 11.8% in those who did not receive remdesivir at 14 days and 12.7% and 15.4%, respectively, at 28 days (Supplementary Table 2). After adjustment for baseline and clinical covariates, the initiation of remdesivir was associated with a significant reduction in all-cause inpatient mortality rate at 14 days (aHR [95% CI], 0.75 [.68–.83]; *P* < .001) and 28 days (0.78 [.72–.86]; *P* < .001; Figure 2*A*).

Similar results were observed in patients with NSOc at baseline (aHR [95% CI], 0.73 [.62–.86] at 14 days and 0.79 [.68–.91] at 28 days) and patients using any supplemental oxygen at baseline (0.75 [.67–.85] and 0.78 [.70–.86], respectively; Figure 2*A* and Supplementary Table 2). In addition, consistent results were obtained in 2 separate sensitivity analyses of using IPTW methodology and assessing remdesivir initiation in the first 2 days of hospitalization vs no remdesivir initiation during the first 2 days of hospitalization.

### Subgroup Analyses

The subgroup analysis indicated that remdesivir was consistently associated with significantly lower 14- and 28-day mortality risks among patients with the different underlying immunocompromising conditions. Specifically, the following results were obtained in subgroups of patients with cancer (including hematological malignancies) (aHR [95% CI], 0.73 [.66–.80] at 14 days and 0.74 [.68–.81] at 28 days), hematological malignancies specifically (0.62 [.52–.74] and 0.64 [.54–.74, respectively), and in SOTRs and HSCT recipients (0.64 [.44–.95] and 0.65 [.48–.87]) (Figure 2*B* and Table 2).

**Table 2.** All-Cause Inpatient Mortality at 14 and 28 Days by Immunocompromising Condition for Patients Hospitalized for COVID-19^a^

Immunosuppressive Condition	14-d Mortality Rate (after IPTW), %		aHR (95% CI)	28-d Mortality Rate (after IPTW), %		aHR (95% CI)
	Nonremdesivir	Remdesivir		Nonremdesivir	Remdesivir	
Cancer (including hematological malignancies)	16.0	12.0	0.73 (.66–.80)	20.1	15.5	0.74 (.68–.81)
Hematological malignancies	15.9	10.3	0.62 (.52–.74)	21.2	14.1	0.64 (.54–.74)
Leukemia	17.9	11.5	0.61 (.47–.79)	21.4	14.7	0.65 (.51–.83)
Lymphoma	13.7	10.6	0.78 (.58–1.04)	20.8	15.2	0.72 (.56–.92)
Multiple myeloma	16.5	7.2	0.39 (.25–.61)	22.0	10.6	0.41 (.28–.61)
SOTRs and HSCT recipients	8.0	5.0	0.64 (.44–.95)	12.2	7.9	0.65 (.48–.87)

Abbreviations: aHR, adjusted hazard ratio; CI, confidence interval; COVID-19, coronavirus disease 2019; d, day; HSCT, hematopoietic stem cell transplant; IPTW, inverse probability of treatment weighting; SOTRs, solid organ transplant recipients.

^a^Data presented in this table are IPTW estimates; hence, sample sizes for the groups are not shown.

For specific types of hematological malignancies, remdesivir was associated with significantly lower 14- and 28-day mortality risks in patients with leukemia (n = 1248; aHR [95% CI] 0.61 [.47–.79] at 14 days and 0.65 [.51–.83] at 28 days) as well as multiple myeloma (n = 607; 0.39 [.25–.61] and 0.41 [.28–.61], respectively) (Figure 2*B* and Table 2). For patients with lymphoma (n = 968), remdesivir was associated with a significantly lower mortality risk at 28 days (aHR [95% CI], 0.72 [.56–.92]) and with a similar beneficial point estimate but not yet reaching statistical significance at 14 days (0.78 [.58–1.04]).

The mean duration of therapy with remdesivir was 4.1 or 4.2 days in all patients and in the different patient subgroups (Supplementary Table 3). The median duration of therapy was 5 days (interquartile range, 3–5 days) across all patient groups.

## DISCUSSION

Our study focuses on the Omicron era through February 2024 and provides current insights regarding the benefit of treating patients with immunocompromising conditions hospitalized for COVID-19 with remdesivir, extending beyond the end of the US public health emergency declaration. Treatment with remdesivir in patients with immunocompromising conditions hospitalized for COVID-19 was associated with reduced all-cause inpatient mortality rates at 14 and 28 days, regardless of severity based on supplemental oxygen requirements. These results build on the previously reported 20%–30% reduction in all-cause inpatient mortality rates with the use of remdesivir in patients with immunocompromising conditions from December 2020 through April 2022, previously reported in this journal [26]. Similarly, a previous single-center retrospective study also concluded that targeted treatment of COVID-19 (including remdesivir) reduced mortality risk in immunocompromised patients during the Omicron period [34].

Our current study and the previously published data from the earlier Omicron period remain the largest data set in patients with immunocompromising conditions hospitalized for COVID-19 [26]. In addition, the 5-day median duration of treatment with remdesivir in this study corresponds to the recommended treatment duration for hospitalized patients not requiring invasive mechanical ventilation or ECMO [22].

### Patients With Cancer

In our study, patients with cancer (including hematological malignancies) who received remdesivir for COVID-19 during hospitalization experienced a 25% lower mortality risk than those with cancer who did not receive remdesivir despite being hospitalized for a primary diagnosis of COVID-19. Overall, the crude proportion of in-hospital mortality risk ranged between 12.0% and 20.1%. These proportions are similar to the previously reported mortality risk of 14.1%–21.5% among patients with cancer (including hematological malignancies) hospitalized for COVID-19 [35, 36]. Previous studies, which report data from early periods of the COVID-19 era, found that the use of remdesivir in patients with cancer was associated with reducing the 28- or 30-day mortality risk by approximately 60% [37, 38]. However, all patients, including those with cancer, had higher mortality risk during the early periods of COVID-19—the pre-Omicron period [35].

Patients with hematological malignancies experienced mortality risks (ranging from 10.3% to 21.2%) similar to those in the larger group of patients with cancer in this study. Although some studies have suggested that patients with hematological malignancies and COVID-19 experience higher all-cause mortality risk than patients with solid tumors, some studies during the Omicron period found similar mortality risks in these 2 patient populations [35, 39, 40]. In our study, the use of remdesivir was associated with >35% reduction in mortality risk in patients with hematological malignancies and >25% reduction in those with any type of cancer. A prominent difference in the mortality risk associated with remdesivir use for COVID-19 in patients with hematological malignancies was observed in patients with multiple myeloma (associated with a 60% lower mortality risk) or leukemia (35% lower). For patients with lymphoma, remdesivir was associated with reduction in mortality risk at 28 days but not at 14 days.

Guidelines on managing COVID-19 specifically in patients with cancer, including hematological malignancies, are primarily limited to smaller regional organizations. The Infectious Diseases Working Party of the German Society for Hematology and Medical Oncology updated their guideline in 2022 to recommend the management of COVID-19 in patients with cancer during the Omicron period [41]. The guideline moderately recommends remdesivir in patients with cancer who are hospitalized with moderate to severe COVID-19 and are not receiving mechanical ventilation or ECMO. Remdesivir is also recommended in combination with other adjuncts such as interleukin 6 receptor or Janus kinase inhibitors (eg, baricitinib) in patients with rapidly progressing COVID-19, even if they were started on mechanical ventilation. The European Conference on Infections in Leukaemia recommends remdesivir as the primary antiviral therapy for patients with hematological malignancies and severe or critical COVID-19 [42].

In mild to moderate COVID-19, remdesivir and nirmatrelvir/ritonavir are antiviral options, as is the viral mutagen molnupiravir. Molnupiravir may accelerate viral evolution, which may be amplified in an immunocompromised population, and has lower efficacy than other antivirals for COVID-19 [42, 43]. As the guideline authors note drug-drug interactions limit the use of nirmatrelvir/ritonavir in patients who would otherwise be expected to benefit [42]. The consensus paper from the European Myeloma Network recommends remdesivir as the only antiviral for the inpatient setting and as one of the antiviral options in the outpatient setting for patients with multiple myeloma [44].

### SOTRs and HSCT Recipients

In our study, the all-cause in-hospital mortality risk was 5%–8%, and an approximately 35% lower mortality risk was associated with remdesivir use among hospitalized SOTRs and HSCT recipients treated with remdesivir for COVID-19. Qualitatively, this mortality risk is lower than previously reported mortality risks, ranging from 18% to 40% in hospitalized SOTRs with remdesivir use [45–48]. However, previous studies focused on early COVID-19 variant periods, had small sample sizes and differed in the types of solid organs transplanted. Further comparisons are complicated by both vaccination and recovery from prior infection. There is a paucity of studies in patients with HSCT.

The American Society of Transplantation and the NIH separately recommended remdesivir as the first-line therapy in outpatient transplant recipients despite the need for intravenous administration [9, 49]. Both organizations highlight the freedom from drug-drug interactions as the main advantage of remdesivir over other antiviral therapies for COVID-19, such as nirmatrelvir/ritonavir [9, 49, 50]. The retired NIH guideline deferred to the recommendations for nontransplant patients when managing COVID-19 in hospitalized SOTRs or HSCT recipients or patients with cancer [9]. The American Society of Transplantation recently removed COVID-19–related recommendations from its website and is currently updating the materials [51].

### Barriers to Appropriate Antiviral Prescribing in Patients With Immunocompromising Conditions Hospitalized for COVID-19

Several barriers remain for appropriate antiviral utilization. A survey of Australasian infectious diseases (ID) specialists concluded that even ID specialists have varied approaches to managing COVID-19 in immunocompromised patients [52]. One explanation for this could be the inconsistencies across different guidelines. The NIH guideline provided guidance on managing COVID-19 in hospitalized patients who are immunocompromised and incorporated real-world evidence into its recommendations [9]. On the other hand, other international and national guidelines for COVID-19 lack information on this topic, provide only general commentary regarding the immunocompromised population, discuss only outpatient management of COVID-19, use strictly randomized clinical trials for their recommendations, or have not been updated with recent evidence [18–21].

Considering the continued risk of COVID-19 in this patient population, it is critical for the international and national guidelines, which are heavily consulted by both generalist and specialist practitioners, to incorporate comprehensive sections on managing COVID-19 in immunocompromised patients. The NIH updated its COVID-19 guideline for the last time on 29 February 2024 and deactivated the living guideline website on 16 August 2024 [9]. Thus, the only comprehensive guideline that addressed management of COVID-19 in immunocompromised patients is neither up to date nor available for practitioners. Although smaller and regional organizations released guidance or consensus statements on managing COVID-19 in different types of immunocompromising conditions, the majority of practitioners may not be familiar with these organizations and their guidelines [41, 42, 44]. ID organizations that are known to all practitioners, such as the Infectious Diseases Society of America, the World Health Organization, and the European Society of Clinical Microbiology and Infectious Diseases, should consider incorporating comprehensive guidance for managing COVID-19 in immunocompromised patients by incorporating all the available evidence to date.

Our large database study provides patient-centered outcomes in a vulnerable population that has been understudied by randomized clinical trials. The study presented most recent data that spanned both earlier and later Omicron periods. The study cohort represented patients across the clinical care spectrum and included patients from rural and urban settings, teaching and community hospitals, and all regions in the United States. The use of PS matching not only accounted for a variety of covariates but also, together with multivariate analysis, minimized confounding. Comprehensive sensitivity analyses, including the IPTW methods, confirmed the robustness of results by yielding consistent results across primary outcome and subgroup analyses.

Conversely, the source database did not include information on time since symptom onset, time since first positive test, or vaccination status. To ensure comparison across patients with similar levels of COVID-19 severity, analyses were stratified by baseline supplemental oxygen requirements. To attempt to account for differences in vaccination status and type of COVID-19 vaccinations received, patients were matched by age group and variant period with preferential matching within the same hospital. This approach is considered to reduce differences due to regional practice patterns, patient attitude toward vaccinations, and availability of vaccines. In addition, data on treatments administered for COVID-19 before hospitalization were unavailable in this data source. Nonetheless, it is plausible to infer that the decision to use remdesivir upon hospitalization is independent of prior outpatient therapy.

As all patients included in this study were already hospitalized for COVID-19, this reflects a failed protection from prior immunity; hence, any biases introduced by the inclusion of such patients in the analyses would be reduced. In addition, it could be expected that the PS-matching approach that led to the balancing of the measured variables in this study (specifically age and key comorbid conditions) is likely to have (at least partially) balanced out unmeasured variables such as vaccination and prior infection as well. Despite this, clinical decision making could be affected as clinicians may be more likely, for example, to prescribe remdesivir to those who are unvaccinated, which would serve to underestimate the benefit of remdesivir observed in this study, as such patients would likely have worse outcomes unrelated to remdesivir administration.

Furthermore, the subgroup analysis for transplant recipients combined data for SOTRs and HSCT recipients, but these 2 patient populations may differ in their levels of immunosuppression especially with more time since transplantation. Moreover, in our study, patients with HIV represented a small percentage of all patients with immunocompromising conditions. Finally, the use of baseline supplemental oxygen was identified using billing charges for supplemental oxygen. To account for hospitals that include the charges for supplemental oxygen supply in the room charges, patients admitted to hospitals that did not report separate charges for supplemental oxygen were excluded from the study.

In conclusion, our large data set revealed up to 25% lower all-cause mortality risk associated with timely clinical initiation of remdesivir in patients hospitalized for COVID-19 during the Omicron period and with immunocompromising conditions present on admission, regardless of the need for supplemental oxygen. The all-cause mortality reduction was observed across all subgroups of patients with immunocompromising conditions, including patients with cancer or hematological malignancies specifically (including leukemia, lymphoma, and multiple myeloma), and SOTRs and HSCT recipients.

To harmonize and optimize the management of patients with immunocompromising conditions hospitalized for COVID-19 across clinical settings, it is essential to incorporate the most recent evidence garnered from routine clinical practice into major ID guidelines. Considering the lack of randomized controlled trials in these vulnerable populations, emerging real-world robust evidence from a large database fills the gap by providing effectiveness of therapeutic interventions in patients hospitalized for COVID-19.

## Supplementary Data

Supplementary materials are available at *Clinical Infectious Diseases* online. Consisting of data provided by the authors to benefit the reader, the posted materials are not copyedited and are the sole responsibility of the authors, so questions or comments should be addressed to the corresponding author.

## Supplementary Material

ciae510_Supplementary_Data

## Notes

***Acknowledgments.*** Medical writing support was provided by Janna Afanasjeva, PharmD, of Certara.

***Authors contributions.*** All authors contributed substantially to the manuscript and approved the final submitted version.

***Data availability.*** The data supporting this study's findings are available from Premier (https://www.premierinc.com/). Restrictions apply to the availability of these data, which were used under license for this study.

***Ethical Approval and Consent.*** This study did not require ethical approval or informed consent. The analysis of data from the US PINC AI Healthcare Database was conducted under an exemption from institutional review board oversight for US-based studies using deidentified healthcare records that are not reidentifiable.

***Financial support.*** This work was supported by Gilead Sciences Inc.

***Supplement sponsorship.*** This article appears as part of the supplement “Harnessing Real-world Data to Inform Clinical Practice: Lessons From the Evolving COVID-19 Era,” sponsored by Gilead Sciences, Inc.

***Potential conflicts of interest***. E. M. and M. B. report employment by and being stockholders with Gilead Sciences during the conduct of this study. A. C. reports funding for study and medical writing provided to their institution (Certara) from Gilead Sciences during the conduct of the study. R. L. G. reports serving on scientific advisory boards for AbbVie, AstraZeneca, Eli Lilly, Gilead Sciences, GSK, Roche, Johnson & Johnson (coronavirus disease 2019 [COVID-19]–related randomized clinical trial, coordinating principal investigator), and Kinevant Sciences (academic steering committee, study investigator; fees to Baylor Scott & White Research Institute); serving as a consultant for Gilead Sciences (honoraria for lectures), Johnson & Johnson, and Kinevant Sciences (through his institution); serving on a speaker bureau for Pfizer unrelated to COVID-19; a gift-in-kind to his institution from Gilead Sciences to facilitate a multicenter clinical trial outside the scope of COVID-19; a de minimis investment in AbCellera; grants or contracts as a study investigator (fees to the Baylor Scott & White Research Institute) from Regeneron, Eli Lilly, Gilead Sciences, Pfizer, Johnson & Johnson, AstraZeneca, and Roivant Sciences (Kinevant Sciences); and receipt of travel support for original scientific presentations from Gilead Sciences. C. C.-M. reports payment or honoraria for lectures/speaking from AstraZeneca, Boehringer Ingelheim, and aTyr Pharma; consulting fees from AstraZeneca; and participation on an advisory board for Gilead Sciences. A. N. A. reports being a principal investigator or coinvestigator of clinical trials sponsored by the National Institute of Allergy and Infectious Diseases, National Institutes of Health, NeuroRx Pharma, Pulmotect, Blade Therapeutics, Novartis, Takeda, Humanigen, Eli Lilly, PTC Therapeutics, OctaPharma, Fulcrum Therapeutics, and Alexion and reports being a speaker and/or consultant for Pfizer, Salix, Alexion, AstraZeneca, Bayer, Ferring, Seres, Spero, Eli Lilly, Novo Nordisk, Gilead Sciences, Renibus, GSK, Dexcom, Reprieve, HeartRite, and Aseptiscope, unrelated to the current study. P. E. S. reports research grants from Gilead Sciences and ViiV for human immunodeficiency virus clinical trials and consulting fees from Gilead Sciences, Merck, and ViiV. A. C. K. reports grants from the National Institutes of Health Adaptive COVID-19 Treatment Trial.
