# Supplementary material for: Remdesivir-Associated Survival Outcomes Among Immunocompromised Patients Hospitalized for COVID-19: Real-world Evidence From the Omicron-Dominant Era
Source: Clin Infect Dis. 2024 Oct 15;79(Suppl 4):S149–59. doi: 10.1093/cid/ciae510 (PMC11638779; doi:10.1093/cid/ciae510)
Supplement: ciae510_Supplementary_Data [file ciae510_supplementary_data.docx]

**Remdesivir-Associated Survival Outcomes Among Immunocompromised Patients Hospitalized for COVID-19: Real-world Evidence From the Omicron-Dominant Era**

Essy Mozaffari^1^, Aastha Chandak^2^, Robert L Gottlieb^3,4,5,6^, Chidinma Chima-Melton^7^, Mark Berry^1^, Alpesh N Amin^8^, Paul E Sax^9^, Andre C Kalil^10^

*^1^Medical Affairs, Gilead Sciences, Foster City, California, USA*

*^2^Evidence and Access, Certara, New York, New York, USA*

*^3^Department of Internal Medicine, Baylor University Medical Center, Dallas, Texas, USA*

*^4^Baylor Scott & White Heart and Vascular Hospital, Dallas, Texas, USA*

*^5^Baylor Scott & White The Heart Hospital, Plano, Texas, USA*

*^6^Baylor Scott & White Research Institute, Dallas, Texas, USA*

*^7^ Pulmonary Division, Tele-ICU, Los Angeles, California, USA*

*^8^Department of Medicine, School of Medicine, University of California Irvine, Irvine, California, USA*

*^9^Division of Infectious Diseases, Brigham and Women’s Hospital, Boston, Massachusetts, USA*

*^10^Division of Infectious Diseases, Department of Internal Medicine, University of Nebraska Medical Center, Omaha, Nebraska, USA*

**Corresponding author:**

Andre Kalil

Department of Internal Medicine, Division of Infectious Diseases

University of Nebraska Medical Center

985400 Nebraska Medical Center

Omaha, NE 68198, USA

Email: [akalil@unmc.edu](mailto:akalil@unmc.edu)

**SUPPLEMENTARY TABLES AND FIGURES**

# Supplementary Table 1. Definitions of Key Study Variables

| Key study variables | | Definitions |
| --- | --- | --- |
| Immunocompromising conditions | Cancer | ICD-10-CM diagnosis codes: C00-C96 |
|  | Hematologic malignancy | ICD-10-CM diagnosis codes: C81.x, C82.x, C83.x, C84.x, C85.x, C88.x, C90.x, C91.x, C92.x, C93.x, C94.x, C95.x, C96.x |
|  | Leukemia | C91.x, C92.x, C93.x, C94.x, C95.x |
|  | Lymphoma | C81.x C82.x, C83.x, C84.x, C85.x |
|  | Multiple myeloma | C90.x |
|  | Solid organ/hematopoietic stem cell transplant recipients | ICD-10-CM diagnosis codes: Z94.x |
|  | Moderate or severe primary immunodeficiencies (example, severe combined immunodeficiencies, Ataxia-telangiectasia, Chediak-Higashi syndrome, Chronic granulomatous disease, Neutropenia) | ICD-10-CM diagnosis codes: D80.x, D81.x, D82.x, D83.x, D84.x, D86.x, D89.0, D89.1, D89.2, D89.3, D89.4x, D89.81, D89.82, D89.89, D89.9, G11.3, E70.330, D71.x, D70.x |
|  | Immunosuppressive medications (example, current use of systemic steroids, immunomodulator, immunosuppressant, chemotherapeutic agent, myelosuppressive agent) | ICD-10-CM diagnosis codes: Z79.52, Z79.60, Z79.61, Z79.62x, Z79.63x, Z79.64, Z79.69, Z79.81x |
|  | Asplenia | ICD-10-CM diagnosis codes: Q89.01, Z90.81 |
|  | Bone Marrow Failure/Aplastic anemia | ICD-10-CM diagnosis codes: D61.x |
|  | HIV | ICD-10-CM diagnosis codes: B20 |
|  | Toxic effects of antineoplastics | ICD-10-CM diagnosis codes: T45.1x |
| Remdesivir use |  | Billing for treatment: Remdesivir;  ICD-10 procedure codes (effective Aug 1, 2020): XW033E5, XW043E5 |
| Comorbidities | Obesity | ICD-10-CM diagnosis codes: E66, Z68.25, Z68.26, Z68.27, Z68.28, Z68.29, Z68.3x, Z68.4x4x |
|  | COPD | ICD-10-CM diagnosis codes: I27.8, I27.9, J40, J41.x, J42, J43.x, J44.x, J45.x, J47.x, J60, J61, J62.x, J63.x, J64, J65, J66.x, J67.x, J68.4, J70.1, J70.3 |
|  | Cardiovascular disease (including hypertension) | ICD-10-CM diagnosis codes: I00.x-I99.x |
|  | Diabetes mellitus | ICD-10-CM diagnosis codes: E10.x-E14.x |
|  | Renal disease | ICD-10-CM diagnosis codes: I12.0, I13.1, N03.2, N03.3, N03.4, N03.5, N03.6, N03.7, N05.2, N05.3, N05.4, N05.5, N05.6, N05.7, N18.x, N19, N25.0, Z49.0, Z49.1, Z49.2, Z94.0, Z99.2 |
| Supplemental oxygen  requirements | IMV/ECMO | Billing charges: invasive mechanical ventilation, tracheostomy, endotracheal tube, intubation, extracorporeal membrane oxygenation |
|  | HFO/NIV | Billing charges: negative-pressure ventilation, positive-pressure ventilation, CPAP, BiPAP, high flow system via nasal cannula, venturi face mask, rebreather, non-rebreather mask, positive expiratory pressure |
|  | LFO | Billing charges: Simple face mask, oxygen pendant, low flow system via nasal cannula, oxygen supply |
|  | NSOc | No billing charges for IMV/ECMO, HFO/NIV, or LFO at baseline (defined above) |
| Admitting diagnosis | Sepsis | Admission diagnosis record with ICD-10-CM diagnosis codes: A02.1, A32.7, A40, A41, A42.7, A54.86, B37.7, R65.20, R65.21, T81.44X |
|  | Respiratory failure/Hypoxemia or Pneumonia | Admission diagnosis record with ICD-10-CM codes: pneumonia (J13, J14, J12.0, J12.1, J12.2, J12.3, J12.9, J15.1, J15.4, J15.6, J15.7, J15.8, J15.9, J16.8, J18.0, J18.1, J18.2, J18.8, J18.9, J11.0, J12.81, J12.82, J12.89, J15.211, J15.29) or acute respiratory distress with hypoxia (J80.X, J96.00, J96.01, J96.90, J96.91, J96.20, J96.21) or hypoxemia (R09.02) |
| Medications | Anticoagulants | Billing for treatment: apixaban, argatroban, desirudin, lepirudin, dabigatran, danaparoid, edoxaban, tinzaparin, heparin (excluding use of heparin flush), ardeparin, bivalirudin |
|  | Baricitinib | Billing for treatment: Baricitinib;  ICD-10-PCS codes (effective Jan 1, 2021): XW0DXM6, XW0H7M6, XW0G7M6 |
|  | Corticosteroids | Billing for treatment: prednisone, prednisolone, methylprednisolone, hydrocortisone, dexamethasone, |
|  | Convalescent plasma | Billing for treatment: convalescent plasma;  ICD-10-PCS codes (effective Aug 1, 2020): XW13325, XW14325 |
|  | Tocilizumab | Billing for treatment: tocilizumab;  ICD-10-PCS codes (effective Aug 1, 2020): XW033H5, XW043H5 |

Abbreviations: BiPAP, bilevel positive airway pressure; CPAP, continuous positive airway pressure; ECMO, extracorporeal membrane oxygenation; HFO, high-flow oxygen; HIV, human immunodeficiency virus; IMV, invasive mechanical ventilation; LFO, low-flow oxygen; NIV, noninvasive ventilation; NSOc, no supplemental oxygen charges

# Supplementary Table 2. Mortality Rates in Remdesivir and Non-remdesivir Patient Cohorts, Overall and According to Baseline Supplemental Oxygen Requirement

|  | **14 days** | | | **28 days** | | |
| --- | --- | --- | --- | --- | --- | --- |
|  | **Non-remdesivir** | **Remdesivir** | **Adjusted HR (95% CI)** | **Non-remdesivir** | **Remdesivir** | **Adjusted HR (95% CI)** |
| **Overall** | 1044 (11.8%) | 813 (9.2%) | 0.75 (0.68-0.83) | 1362 (15.4%) | 1120 (12.7%) | 0.78 (0.72 - 0.86) |
| **Baseline supplementary oxygen requirement** | | | | | | |
| **No supplementary oxygen charges** | 365 (7.6%) | 276 (5.8%) | 0.73 (0.62 - 0.86) | 462 (9.7%) | 380 (8.0%) | 0.79 (0.68 - 0.91) |
| **Any supplemental oxygen charges** | 679 (16.8%) | 537 (13.3%) | 0.75 (0.67 - 0.85) | 900 (22.2%) | 740 (18.3%) | 0.78 (0.70 - 0.86) |

Abbreviations: CI, confidence interval; HR, hazard ratio

# Supplementary Table 3. Duration of Remdesivir Treatment in Matched Patients with Immunocompromising Conditions Hospitalized for COVID-19

| **Matched patient cohort** | **Mean (SD), days** | **Median (IQR), days** |
| --- | --- | --- |
| All patients with immunocompromising condition (N=8822) | 4.1 (1.4) | 5.0 (3.0; 5.0) |
| Cancer (n=3781) | 4.1 (1.4) | 5.0 (3.0; 5.0) |
| Hematologic malignancy (n=1456) | 4.2 (1.4) | 5.0 (3.0; 5.0) |
| Leukemia (n=654) | 4.2 (1.4) | 5.0 (3.0; 5.0) |
| Lymphoma (n=476) | 4.2 (1.4) | 5.0 (3.0; 5.0) |
| Multiple myeloma (n=321) | 4.1 (1.4) | 5.0 (3.0; 5.0) |
| Solid organ/hematopoietic stem cell transplant recipients (n=696) | 4.2 (1.4) | 5.0 (3.0; 5.0) |

Abbreviations: COVID-19, coronavirus disease 2019; IQR, interquartile range; SD, standard deviation

# Supplementary Figure 1. Balance of Standardized Mean Difference Before and After Propensity Score Matching Among Patients with Immunocompromising Conditions Hospitalized for COVID-19


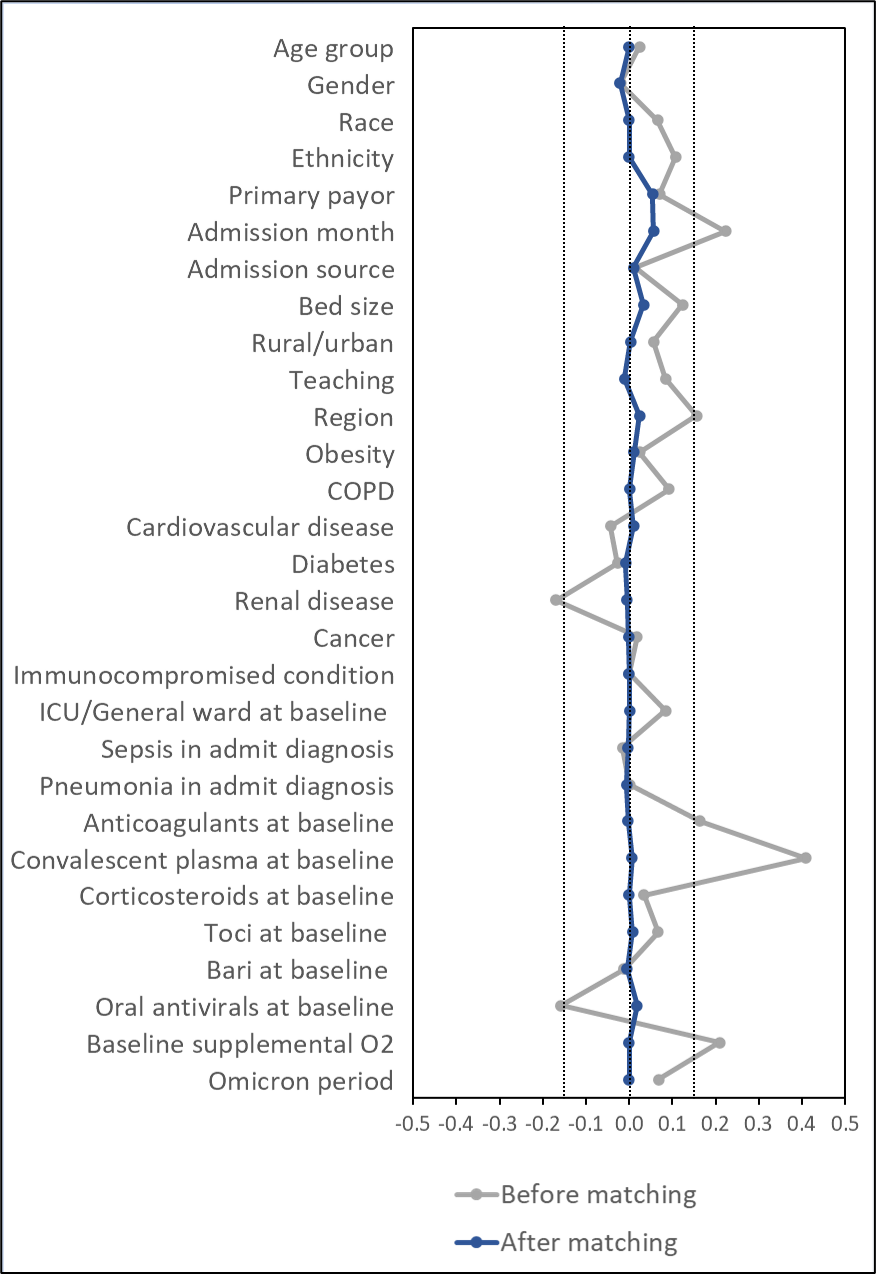


Abbreviations: CCI, Charlson Comorbidity Index; COPD, chronic obstructive pulmonary disorder; ICU, intensive care unit; IPTW, inverse probability of treatment weighting; O2, oxygen.
